# Efficacy of Dog Training With and Without Remote Electronic Collars vs. a Focus on Positive Reinforcement

**DOI:** 10.3389/fvets.2020.00508

**Published:** 2020-07-22

**Authors:** Lucy China, Daniel S. Mills, Jonathan J. Cooper

**Affiliations:** Animal Behaviour, Cognition and Welfare Research Group, School of Life Sciences, University of Lincoln, Lincoln, United Kingdom

**Keywords:** dog training, dog welfare, electronic collar, reinforcement, punishment

## Abstract

We assessed the efficacy of dog training with and without remote electronic collars compared to training with positive reinforcement. A total of 63 dogs with known off-lead behavioral problems such as poor recall were allocated to one of three training groups (each *n* = 21), receiving up to 150 min of training over 5 days to improve recall and general obedience. The 3 groups were: E-collar—manufacturer-nominated trainers who used electronic stimuli as part of their training program; Control 1—the same trainers following practices they would apply when not using electronic stimuli; and Control 2—independent, professional trainers who focused primarily on positive reinforcement for their training. Data collection focused on dogs' response to two commands: “Come” (recall to trainer) and “Sit” (place hindquarters on ground). These were the two most common commands used during training, with improving recall being the target behavior for the subject dogs. Measures of training efficacy included number of commands given to elicit the response and response latency. Control 2 achieved significantly better responses to both “Sit” and “Come” commands after a single instruction in the allocated time. These dogs also had shorter response latencies than the E-collar group. There was no significant difference in the proportion of command disobeyed between the three groups, although significantly fewer commands were given to the dogs in Control 2. There was no difference in the number of verbal cues used in each group, but Control 2 used fewer hand and lead signals, and Control 1 made more use of these signals than E-collar group. These findings refute the suggestion that training with an E-collar is either more efficient or results in less disobedience, even in the hands of experienced trainers. In many ways, training with positive reinforcement was found to be more effective at addressing the target behavior as well as general obedience training. This method of training also poses fewer risks to dog welfare and quality of the human-dog relationship. Given these results we suggest that there is no evidence to indicate that E-collar training is necessary, even for its most widely cited indication.

## Introduction

Successful obedience training of dogs requires effective use and timing of cues (often referred to as “signals”) alongside reinforcement and/or punishment by dog trainers. Where dog training involves aversive or noxious stimuli, this can lead to punishment if dogs do not behave as desired (1, 2). A growing understanding of the application of learning theory to dog welfare has led many training organizations, welfare charities and academics to advocate what they consider to be more humane methods, with a greater focus on the use and timing of rewards (3–9).

Electronic training aids take a number of forms, but they commonly involve a collar-born device (E-collar) which can deliver a static electric stimulus to the dog's neck as well as a number of other stimuli, such as auditory or haptic/vibration signals (10). Collar-born devices include: remote, hand-operated devices; bark- or noise-activated control collars; and containment systems (or invisible fencing) (11). Generally, collars are designed to allow the auditory/haptic signals to be paired with the delivery of the electric stimulus as a form of “warning” cue. If the dog ignores this, the electric stimulus may be applied until the desired behavior is performed. In this way dogs may learn through a combination of negative reinforcement and classical conditioning to avoid the electric stimulus by performing the desired response, however, if the delivery of electric stimuli is poorly timed or inescapable, then undesirable associations may be formed (11–13). Opponents of E-collars have argued that because these devices use aversive stimuli to deter undesirable behavior, they pose an increased risk of undesirable training outcomes (such as negative changes in affective state or unanticipated associations) compared to reward-focused training, especially in the hands of poorly trained or inexperienced owners (14–18). In contrast, those who advocate the use of remote E-collars have argued that the devices, especially in the hands of experienced trainers, can be used as to modify behavior through negative reinforcement, with limited exposure to noxious stimuli, so are a valuable training aid. Collar manufacturers suggest that an advantage of these systems is that they give handlers control over a dog even at distance (19), and effectively suppress highly motivated behaviors, such as predatory behavior; a cause of livestock chasing or unintentional killing of wildlife (20–23). It has also been claimed that where E-collars are successful in treating behavioral problems, dogs may avoid unnecessarily euthanasia, an outcome that would be distressing to the owners (24).

The use of E-collars in dog training appears to be declining in the UK, from an estimate of 6% of all pet dogs in 2012 (25) to around 1% in 2019 (26). This decrease may reflect current government policy on the devices in Wales [devices banned under the (27)] and Scotland [not condoned in dog training and use may lead to punishment (28)], with restrictive legislation proposed for England (29) as well as high-profile campaigns against their use [e.g., by the (18)]. Nevertheless, these figures while appearing relatively low still suggest about 100,000 dogs in the UK are subject to E-collar use, and these devices remain legal in many other countries.

Research studies are cited selectively by both advocates and opponents of E-collars to support their claims, often with insufficient appreciation of the quality of experimental design or with a biased evaluation of evidence, such as the multiple possible interpretations of isolated behavioral indicators of welfare (11). However, the necessity of these devices [which has been used to justify their continued use e.g. (30)] depends on their efficacy compared to other training approaches (11, 31). Efficacy can be assessed objectively using specific target behavioral measures, and the use of professionally designed regimes delivered by experienced trainers can reduce the risk of sampling bias. In the current study we directly assessed the efficacy of the use of electronic collars to improve recall (the target behavior) and general obedience in dogs compared to training without E-collars. Dogs used in this study were referred to experienced, professional trainers as their owners had been experiencing significant obedience problems, including poor recall, but also chasing livestock and/or aggressive behavior to other dogs. The current study focussed on remote, hand-operated devices, as these were the most commonly used form in the UK at time of study (25, 32); being primarily used as a means of discouraging chasing behavior and improving recall. We used training records collected during DEFRA funded research (33) on behalf of the UK government. In contrast to the previously published work (31), where efficacy was assessed by owner feedback, this study recorded the speed and reliability of response after each command, in order to derive a more rigorous, systematic and objective measure of efficacy.

## Materials and Methods

Data were extracted from dog training videos, which were originally recorded as part of a DEFRA funded study (33) collected over a period of 6 months in 2010/11. Details of the recruitment of dogs, the training regimes and video data collection have previously been published (31, 33), so the methods presented here provide an overview with additional details of differences in the approach taken in the current study.

Ethical approval was provided by the University of Lincoln Research Ethics Committee. Owners and trainers that participated in the study gave their informed consent for the use of their dogs and video recordings in the study. Home Office Inspectorate were consulted, and indicated that the work did not constitute a procedure and consequently a Home Office License would not be required based on the following conditions: E-collar use was legal in England and Scotland at the time of the study; dogs were being referred for behaviors commonly associated with E-collar use in the UK; the training was being conducted by experienced professional trainers using normal training programmes with the informed consent of owners.

### Training Groups

All dogs used in this study had been referred for behavioral concerns including poor recall and livestock worrying and owners had been recommended to seek professional training to resolve those problems. The 63 dogs involved in the study were all older than 9 months of age and had no prior experience with electronic collars. Dogs in E-collar and Control Group 1 were trained in Autumn/Winter 2010 and were randomly allocated to their training group. Dogs in Control Group 2 were trained in Spring 2011, meaning subjects could be recruited to match the dogs trained with E-collars on the basis of referred behavioral problem and owner's assessment of severity. The 3 training groups were as follows:

- E-collar Group (EC: *n* = 21): dogs were trained using active electronic collars to improve recall and general obedience by experienced, manufacturer-nominated trainers (ECMA) (chosen to represent best-practice use of the E-collar). Trainers followed approved practice as recommended by ECMA, including assessing the dog's sensitivity to electric stimuli prior to training, and pairing vibration cue with the electric signal with the aim of modifying behavior through negative reinforcement. Dogs in this group also experienced positive reinforcement, such as rewarding dogs with food and negative reinforcement such as lead pressure.
- Control Group 1 (C1: *n* = 21): dogs were trained by the same trainers who worked with the E-collar group, using a mix of food rewarded positive reinforcement and negative reinforcement such as lead pressure to improve recall and general obedience but without use of electronic stimuli;
- Control Group 2 (C2: *n* = 21): dogs were trained to improve recall and general obedience by experienced professional trainers who were members of Association of Pet Dog Trainers (APDT UK); an organization which does not support the use of E-collars in dog training (chosen to represent best-practice use of positive reinforcement or “reward-based training”).

The dog population used in this study was broadly similar to the populations described by Blackwell et al. (25) in their survey of use of electronic training aids, and there were no significant differences between the dogs allocated to the three treatments in type of dog or reason for referral (31). Gundogs (25%), cross-breeds (25%), pastoral (17%) and terriers (13%) were the most commonly represented breed types with similar numbers in each treatment group, whereas there were no dogs from toy or utility breed groups. 34 (54%) dogs were female, with 21 (33%) of these neutered and 13 (21%) entire female dogs. Of the 29 male dogs, there was also a slightly higher number of neutered dogs (19, 30% of total population) than entire male dogs (10 or 16%); however there were no significant gender biases between the treatment groups. Chasing was the most common reason for referral in the study population (51 out of 63 dogs or 81% of population), representing 18 dogs in the E-Collar Group, 17 dogs in Control Group 1 and 16 dogs in Control Group 2. Sheep or lambs were the most commonly cited chase target, where owners reported chasing as a problem behavior, although owners also listed other livestock such as horses and poultry, wildlife such as rabbits or squirrels, as well as cars and joggers as targets for chasing. The remaining dogs had either been referred for poor recall (9 dogs of which 1 was in E-collar Group and 4 each in Control Group) or aggressive interactions with other dogs whilst off lead (3 dogs, 2 of which were in E-collar Group, 1 in Control Group 2 and none in Control Group 1). The majority of owners described their dogs as exhibiting the referred behavior “Always” (31 dogs or 49% of population), or “Frequently” (24 dogs or 38% of population indicating the high severity as perceived by owners. When these two ratings were pooled, there was no difference in owner assessment of severity between the three groups (31).

All dogs in the study wore an E-collar during training sessions in order for data analyzers to be blind to training group during video observation. Dogs in the Control groups wore a de-activated or “dummy” collar, whilst the e-collars worn by dogs in E-collar group were active and useable by the trainers. Training mainly occurred in field locations, with penned sheep, penned chickens and other (on lead) dogs, as potential distractors during training. Dogs were primarily kept on 10 m long leads throughout training session; however, trainers had the option to drop the lead or remove the lead from the dog when considered appropriate. During training dogs were normally within 1 m of the trainer (around 70% of time in all three groups) with <5% of time spent more than 5 m distant from trainer (in all three groups). Trainers in all groups had access to food rewards and could use them as the trainer deemed appropriate during training. Previous work (31) had indicated that whilst dogs in Control Group 2 received fewer signals per 15 min training session than dogs in E-collar Group or Control Group 1 (32 signals compared with 59 and 56, respectively), they were much more likely to receive food reward following a successful response, than dogs in Control Group 1 or E-collar group. Preliminary observations prior to this study to determine which commands were most common in the three groups confirmed these previous reports with food estimated to be used about 5 times more frequently as a reward during training by Control Group 2, than E-collar or Control Group 1 (34). This rate of reward would be consistent with the emphasis on reward based training in Control Group 2, compared to a mix of training approaches in the other treatment groups. Two training sessions were recorded daily (one in the morning and one in the afternoon) for each dog, for up to 5 consecutive days, producing an average of 28.5 ± 4.5 (mean ± SD) minutes of video record per dog per day, and up to 150 min over the 5 training days.

### Data Collection

Data for the current study were taken from the two training sessions on the first, third and fifth day of training for each dog. Measures focused on indicators of efficacy and reliability of obeying command, including latency to complete response and number of commands required to complete desired response. Data collection focused on the two commands that were most commonly used in all 3 training groups, and could be easily distinguished from video data. These were a “Come” command normally used for recall of dogs when at a distance from the trainer and a “Sit” command normally used to require the dog to place its hind-quarters on ground and remain stationary for brief periods of time (See Table 1). “Come” and “Sit” commands were chosen for several reasons. Both commands could be clearly identified and obtained from the videos, across all groups, and could not be confused with other commands. During preliminary analysis of video records, these commands were also found to be the most commonly used in all three training groups. Three forms of signal or mode of delivery of training signals were noted in preliminary observation; verbal, hand and lead, and these are also defined in context in Table 1. We also recorded: if dogs began the recall response after a single “Come” command (Come); if multiple commands (Come+) were used to initiate the recall response; or if the dog did not initiate the response (disobey; see Table 2). Similarly, we recorded: if a sit response was completed after a single signal (Sit); if multiple signals were needed (Sit+); or if the dog did not perform a Sit response to the “Sit” commands (disobey). In preliminary observations, to determine timeframes for these definitions of outcome, most dogs responded within 2 s of initial command, and where dogs were given additional commands, this was normally limited to 2 or occasionally 3 commands within the 10 s of the initial command. Where dogs had not completed the response within 10 s of initial command, trainers normally ceased this sequence of commands and after a brief rest normally longer than 10 s would resume with a new command. This approach was similar across the three training groups, so the definition of successful responses and disobey could be applied to all groups. To control for the different number of commands given, absolute values were converted into % of commands to compare reliability of response between the three groups. Where dogs responded to the “Come” command the latency was recorded as the time from delivery of first command to the dog initiating the recall response, whereas latency to sit was recorded as the time to place hind-quarters on ground following delivery of the first “Sit” command signal.

**Table 1:** Descriptions of the mode of delivery (or signals) for the Come and Sit commands.

**Command given**	**Command type**	**Description**
Come	Verbal	The dog is encouraged to return to the trainer/owner from distance upon the verbal “come” command; noises of encouragement given after this include clicking, whistling, kissing-sounds, etc.; related verbal expressions such as “let's go,” “come on” etc and use of the dog's name
	Hand signal	The dog is encouraged to return to the trainer/owner from distance upon the visual hand signal of a beckoning motion from the arm and hand extended away from the body and the arm of hand is repeatedly drawn toward the body; may also be gestured by the patting of the trainer/owner's leg. May be accompanied by other more physical actions noted at the time
	Lead signal	The dog is encouraged to return to the trainer/owner from distance following a tug on the lead being toward the trainer/owner or the lead is flicked to bring the dog toward the trainer/owner. May be accompanied by other more physical actions noted at the time
Sit	Verbal	The dog is asked to place its rear end on the ground upon issuing of the “sit” command verbally
	Hand signal	The dog is asked to place its rear end on the ground by the hand of the trainer/owner being brought up toward the chest/center of the body or the trainer pointing their finger down over the dog's head. May be accompanied by other more physical actions noted at the time
	Lead signal	The dog is asked to place its rear end on the ground upon the lead being pulled vertically above the dog's head or down toward the ground. May be accompanied by other more physical actions noted at the time

**Table 2:** Description of dogs' responses to “Come” and “Sit” commands.

**Command given**	**Command response**	**Description**
Come	Obeys after first command	The dog correctly responds to the “come” command by taking steps, at any speed, toward the trainer/owner following the first instance of the command being given
	Obeys after repeated commands	The dog correctly responds to the “come” command by taking steps, at any speed, toward the trainer/owner following multiple instances of the command being given
	Disobey	The dog fails to appropriately respond to the “come” command, either by failing to move toward the trainer/owner or no correct response within 10 s of the first command thus acting as a cut-off point for the command
Sit	Obeys after first command	The dog correctly responds to the “sit” command by placing the hind quarters on the ground following the first instance of the command being given
	Obeys after repeated commands	The dog correctly responds to the “sit” command by placing the hind quarters on the ground following the first instance of the command being given
	Disobey	The dog fails to appropriately respond to the “sit” command, either by failing to place the hind quarters on the ground or no correct response occur within 10 s of the first command thus acting as a cut-off point for the command

### Data Extraction and Statistical Analysis

Training videos were viewed in a random order and blinded, such that the viewer could not associate dogs with their respective group, using Solomon Coder software (version: beta 17.03.22). Following collection, raw data was extracted from the Solomon Coder files into a Microsoft Excel spreadsheet, separating each dog into their allocated training group. Data for the number of commands (Table 3) were analyzed per training session. A small number of sessions focused on just recall or just sit, so morning and afternoon sessions were aggregated, for analysis of the percentage of dogs responding to the first signal, multiple signals, or disobeying and for the calculation of latencies. This provided a single daily measure for each dog. Previous work Cooper et al. (31) had indicated no significant differences in dogs' behavior between morning and afternoon sessions, and exploratory comparison of morning and afternoon data in this study was consistent with this.

**Table 3:** Mean number of commands given per training session (±SE) for dogs trained with E-collars and the two control groups, including number of verbal, hand, and lead signals, number of times a single “Come” and “Sit” command were given and numbers of times multiple signals were given for each command (Come+ and Sit+) and the number of times dogs obeyed on first comment, obeyed after multiple commands (Obey+) or did not obey.

**Command given**	**Mean** **±** **Standard Error of commands given**			**F-Ratio from GLM**
	**E-Collar**	**Control 1**	**Control 2**	
Verbal	16.5 ± 1.4	20.5 ± 1.6	16.6 ± 1.1	*F*_(2, 293)_ = 3.05, *P* = 0.051
Hand	5.4 ± 0.4_a_	8.9 ± 0.7_b_	1.6 ± 0.2_c_	*F*_(2, 293)_ = 57.7, *P* < 0.001
Lead	4.2 ± 0.5_a_	7.5 ± 1.0_b_	0.1 ± 0.0_c_	*F*_2, 293)_ = 39.6, *P* < 0.001
Sit	12.5 ± 0.8_a_	16.2 ± 1.0_b_	3.4 ± 0.5_c_	*F*_(2, 293)_ = 69.2, *P* < 0.001
Sit+	3.4 ± 0.4_a_	5.5 ± 0.6_b_	0.6 ± 0.1_c_	*F*_(2, 293)_ = 35.4, *P* < 0.001
Come	7.4 ± 0.6_a_	10.2 ± 0.8_b_	11.8 ± 0.8_b_	*F*_(2, 293)_ = 8.92, *P* < 0.001
Come+	2.9 ± 0.5_a_	4.9 ± 0.7_b_	2.5 ± 0.3_a_	*F*_(2, 293)_ = 6.84, *P* = 0.001
Obey	15.4 ± 1.1_a_	19.2 ± 1.1_b_	12.8 ± 0.9_a_	*F*_(2, 293)_ = 8.78, *P* < 0.001
Obey+	4.1 ± 0.3_a_	6.3 ± 0.5_b_	2.0 ± 0.2_a_	*F*_(2, 293)_ = 37.5, *P* < 0.001
Disobey	0.4 ± 0.1_a_	1.0 ± 1.1_b_	0.4 ± 0.1a	*F*_(2, 293)_ = 9.50, *P* < 0.001

*Different subscripts (a, b, and c) indicate where training groups differed based on Tukey pair-wise comparisons*.

Statistical analysis of the data was conducted using Minitab 17.0, using General Linear Models (GLMs). Training groups and days (1, 3, and 5) were treated as fixed factors, whilst individual dog IDs were random factors nested within the training groups. As the focus was on efficacy outcomes we focused on main effects and did not include interactions within our models, so as not to unnecessarily inflate the degrees of freedom in the models. Unless stated otherwise data is presented as mean ± standard error, since our focus was on differences between groups and not group variability.

## Results

### Number of Commands, Signals, and Responses

On average 20.3 ± 0.6 commands were given per training session, of which 15.7 ± 0.6 (77%) were obeyed on first command, 4.1 ± 0.2 (20%) obeyed after multiple commands and only 0.6 ± 0.1 (3%) disobeyed. On average the number of signals per training session was 26.8 ± 0.8. The majority of signals were verbal with 17.8 ± 0.8 verbal signals per session (66% of all signals). There were 5.2 ± 0.3 hand signals per training session (19% of all signals) and 3.8 ± 0.4 lead signals (14%). There was no difference in the number of verbal signals given to dogs in the 3 training groups (Table 3), but Control Group 1 consistently received more hand and lead signals than dogs trained with E-collars, whilst Control Group 2 consistently had fewer hand and lead signals than the other groups. As a consequence, Control Group 1 received most signals during training, whilst Control Group 2 received fewer signals during the training period than the other groups [*F*_(2, 293)_ = 30.2, *P* < 0.001].

Control Group 2 performed fewer “Sit” responses during training than the E-collar group and Control Group 1, following single commands, following use of multiple commands (Table 3) and overall [*F*_(2, 293)_ = 74.5, *P* < 0.001]. Control Group 1 performed the most “Sit” and most “Come” responses following multiple commands, whilst the E-collar Group performed least “Come” responses following a single command and in total [*F*_(2, 293)_ = 5.51, *P* = 0.005].

Control Group 1 exhibited more disobeys than either the E-collar training group or Control Group 2 (Table 3), but also completed more responses after single and multiple commands as they received most commands of the three training groups. When the percentage of responses was analyzed to account for the different number of commands between the training groups, there was no difference in percentage of disobeys between the three training groups (Table 4). Control Group 2, however, had a higher percentage of performing both “Come” and “Sit” responses on first command and lower percentage following multiple commands than either Control Group 1 or the E-collar Group.

**Table 4:** Mean percentage of “Come” and “Sit” commands (± SE) obeyed after a single signal, obeyed after multiple signals (Obey+) or not obeyed for dogs trained with E-collars and the two control groups.

**Percentage**	**Mean** **±** **Standard Error of commands given**			***F*-Ratio from GLM**
	**E-Collar**	**Control 1**	**Control 2**	
% Obey Come	71.0 ± 3.2_a_	72.4 ± 2.7_a_	82.5 ± 2.3_b_	*F*_(2, 114)_ = 4.46, *P* = 0.015
% Obey+ Come	26.3 ± 2.8_a_	24.4 ± 2.4_a_	15.4 ± 2.2_b_	*F*_(2, 114)_ = 4.33, *P* = 0.017
% Disobey Come	2.7 ± 0.1	3.2 ± 0.01	2.1 ± 0.01	*F*_(2, 114)_ = 0.66, *P* = 0.52
% Obey Sit	76.8 ± 2.8_a_	72.7 ± 2.7_a_	83.5 ± 3.2_b_	*F*_(2, 114)_ = 3.49, *P* = 0.036
% Obey+ Sit	18.9 ± 2.0_a_	21.9 ± 2.1_a_	10.6 ± 2.1_b_	*F*_(2, 114)_ = 6.69, *P* = 0.002
% Disobey Sit	4.4 ± 0.2	5.4 ± 0.2	3.7 ± 0.1	*F*_(2, 114)_ = 0.36, *P* = 0.70

*Different subscripts (a and b) indicate where training groups differed based on Tukey pair-wise comparisons*.

**Figure 1:**
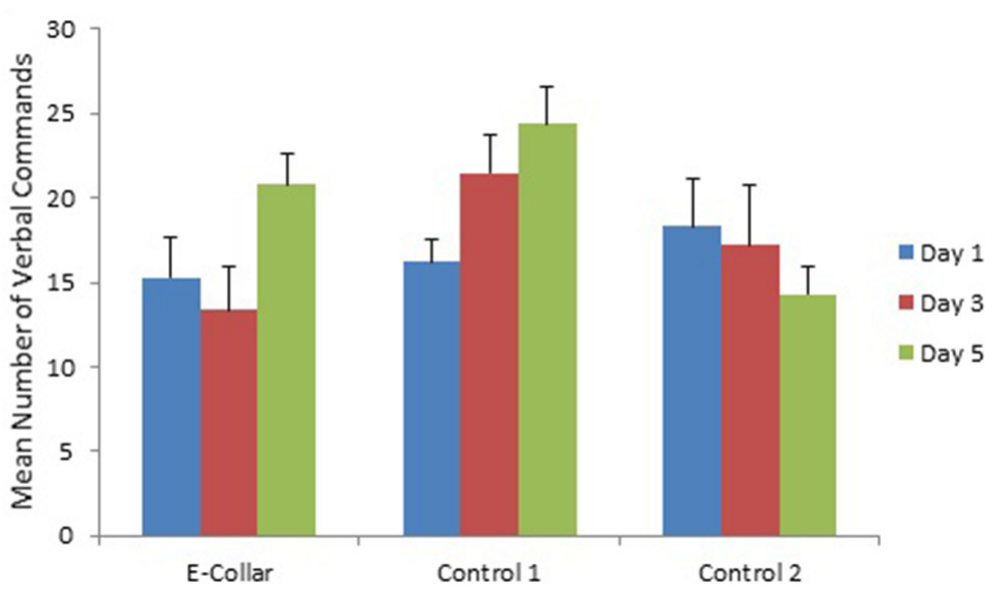
The mean (with SE) number of verbal commands given to dogs in the E-collar training group and the two Control groups over the 3 training days. See Tables 3, 4 for analysis of differences between groups; no significant differences between training days.

**Figure 2:**
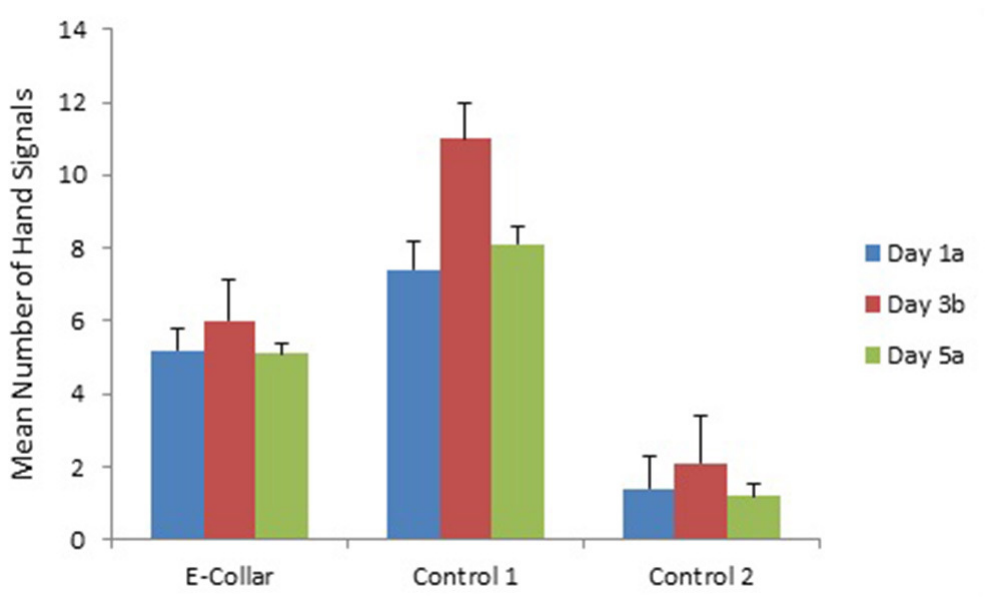
The mean (with SE) number of hand commands given to dogs in each training group over the 3 training days. Subscripts (a and b) indicate where training days differed based on Tukey pair-wise comparisons. See Tables 3, 4 for analysis of differences between groups.

**Figure 3:**
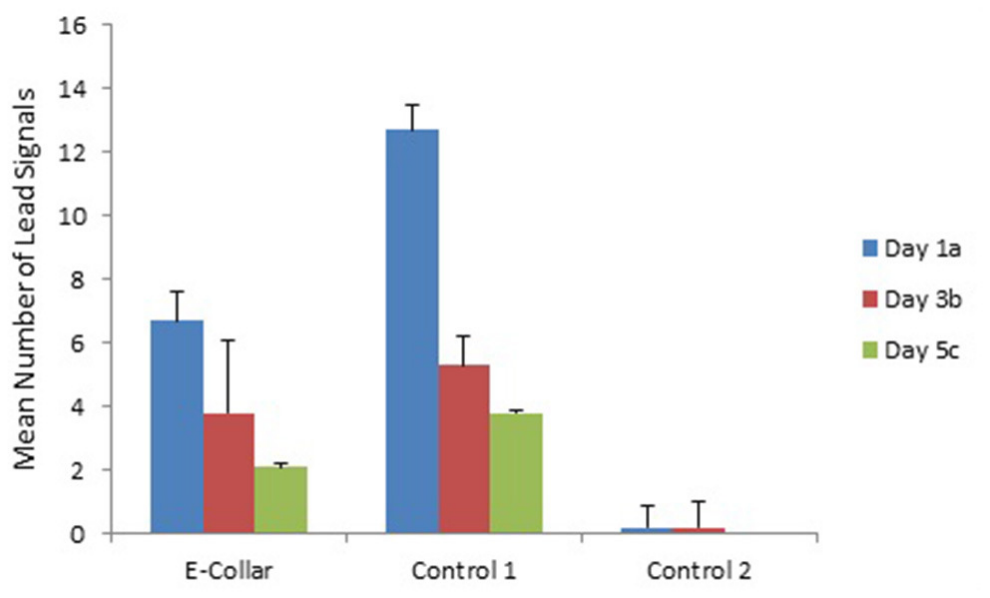
The mean (with SE) number of lead commands given to dogs in the E-collar training group and the two Control groups over the 3 training days. Subscripts (a, b, and c) indicate where training days differed based on Tukey pair-wise comparisons. See Tables 3, 4 for analysis of differences between groups.

Training day had no effect on number of commands or response rate, except for the use of signals (Figures 1–3). Use of lead signals declined from day 1 to day 5 [Figure 3: *F*_(2, 293)_ = 17.5, *p* < 0.001] and use of hands signals was most common on day 3 [Figure 2; *F*_(2, 293)_ = 4.04, *p* = 0.018]. There was however no change in number of verbal signals used over the training days, and overall total number of signals used did not differ across training days [*F*_(2, 293)_ = 0.16, *P* = 0.85].

### Latency to Respond

Overall, the mean latency to respond to the “Come” command was 1.24 ± 0.05 s, whereas dogs took a mean of 1.64 ± 0.06 s to complete the “Sit” commands. There were significant differences in latency to respond to both the “Come” [*F*_(2, 114)_= 5.89; *p* = 0.04] and the “Sit” command [*F*_(2, 101)_= 12.3; *P* < 0.001] between the training groups (Table 5). For the “Come” command there was a shorter latency to respond by Control Group 2 compared with the E-collar Group. The difference in latency to respond to the “Sit” command was largely similar to that of the “Come” command, however Control Group 2 responded sooner than both the E-collar Group and Control Group 1.

**Table 5:** Mean latency to complete response in seconds from initial command (± standard deviation) for those dogs that completed come and sit responses from the E-collar and 2 Control training groups and on different days.

**Command given**	**Latency to respond**			**GLM**
	**E-Collar**	**Control 1**	**Control 2**	
Come	1.35 ± 0.11_a_	1.24 ± 0.09_ab_	1.13 ± 0.05_b_	*F*_(2, 114)_= 5.89, *P* = 0.04
Sit	1.67 ± 0.11_a_	1.81 ± 0.12_a_	1.36 ± 0.11_b_	*F*_(2, 101)_= 12.3, *P* < 0.001
	**Day 1**	**Day 3**	**Day 5**	
Come	1.26 ± 0.10	1.30 ± 0.08	1.14 ± 0.08	*F*_(2, 114)_= 1.82, *P* = 0.17
Sit	1.44 ± 0.10_a_	1.72 ± 0.11_b_	1.76 ± 0.15_b_	*F*_(2, 101)_= 5.61, *P* = 0.005

*Different subscripts (a and b) indicate where training groups differed based on Tukey pair-wise comparisons*.

**Figure 4:**
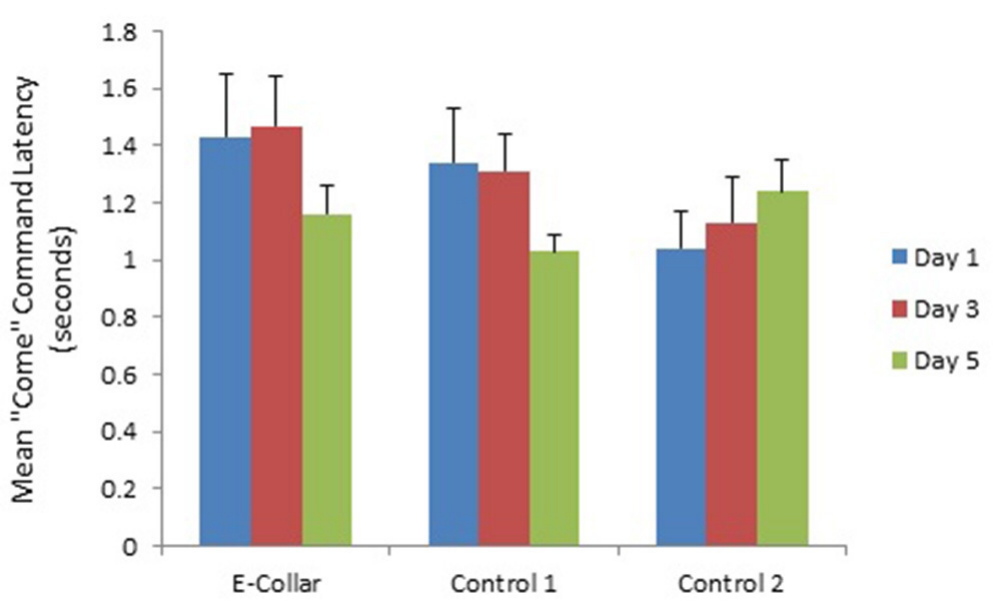
The mean (with SE) latency to respond to “Come” command by dogs in the E-collar training group and the two Control groups over the 3 training days.

**Figure 5:**
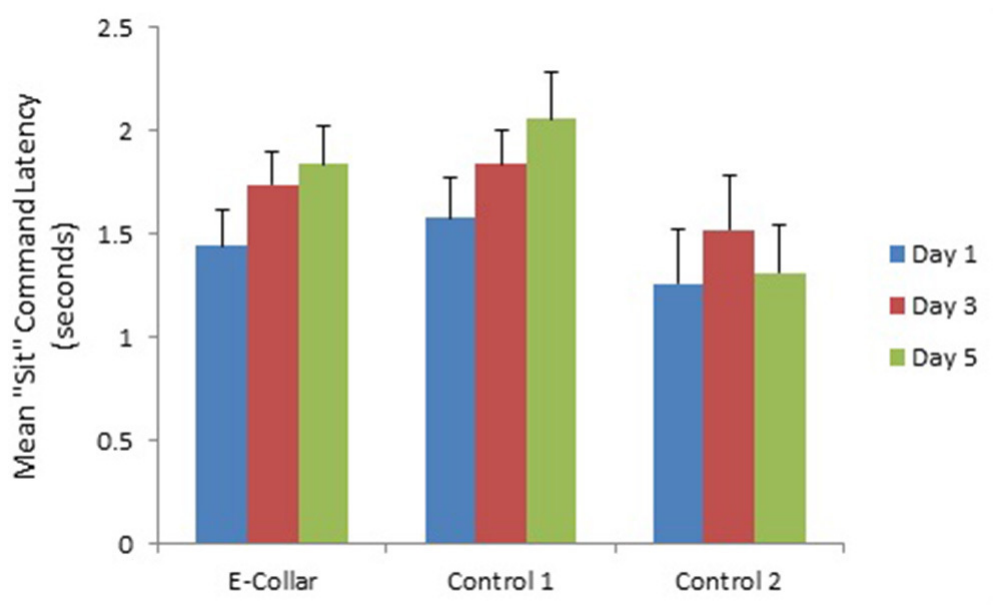
The mean (with SE) latency to respond to “Sit” command by dogs in the E-collar training group and the two Control groups over the 3 training days.

Although the E-collar Group and Control Group 1 appeared to show a decline in latency to respond to the “Come” command over the study period (Figure 4) there was no significant change in latency to come between the 3 training days [*F*_(2, 114)_ = 1.82; *P* = 0.17]. In contrast there was a change in latency to sit [*F*_(2, 101)_ = 5.61; *P* = 0.005] with longer latencies to sit on day 3 and day 5 compared to day 1 (Table 5), which was related to increased latency in the E-collar training group and Control Group 1, as training progressed (Figure 5).

## Discussion

Each of the three training groups had successful training outcomes to both “Come” and “Sit” commands. The proportion of responses that were performed following first command was high in all three groups, and the proportion of disobeys was low throughout the study and did not differ between training groups. These findings are consistent with owner satisfaction with training outcomes as reported previously (31) and should be expected as all trainers were professionals, with extensive experience of training dogs to improve recall and general obedience. The reward-based Control Group 2, however, had a higher proportion of obeys after first command to both “Come” and “Sit” commands and required fewer multiple commands to initiate a recall or complete a sit response. This suggests that the reward-based training was the most effective approach not only for recall which was the target behavior in training, but also for other commands, even though the reward based trainers did not spend as much of their time training on sit command as the other two training groups.

Latencies to respond also indicate successful training outcomes in all three groups with dogs beginning to return to the trainer on average 1.24 s after delivery of a “Come” command and dogs completing the sit response on average 1.64 s after a “Sit” command. The slightly longer latency to sit potentially reflected this measure being based on completion of response, whereas latency to “Come” response was determined from the initiation of recall with dogs beginning to return to trainer. Although differences between groups were small, dogs in Control Group 2, showed a shorter latency to begin to return than the E-collar Group, which is consistent with the higher proportion of responses seen following a single command in this group. There were also differences between the groups in time to complete the sit response, with Control Group 2 being faster to complete this response than both the E-collar group and Control Group 1. This was also consistent with a higher proportion of dogs completing this response after a single command. It is noteworthy that there was little difference in latency to sit between the three groups on the first day of training, as dogs in all three groups had a reliable response to the “Sit” command before training, but longer latencies in the E-collar and Control 1 group become apparent as training progressed. These findings are consistent with the reported public perception that E-collars have lower success rates than reward-based training for recall and chase problems (25), and concerns regarding efficacy of training programs involving potentially aversive stimuli raised by Hiby et al. (3), Rooney and Cowan (4), Fernandes et al. (5), Ziv (6), and Masson et al. (7, 35).

Two factors apart from the use of electric stimuli during training should be explored before drawing conclusions with regard to the efficacy of the three training methods. The first relates to the weather conditions as E-collar Group and Control Group 1 were trained in mid-winter, whereas Control Group 2 were trained 4 months later in early Spring. This was in part due to availability of industry nominated trainers, but also allowed time to select dogs from the larger population available for training without E-collars to best match those referred to E-collar Group. Although there has been no published work on seasonal variation in training outcomes in dogs, there are likely to be variation in environmental conditions, that may impact on these outcomes. Indeed the winter training period in particular featured some extreme weather conditions with lying snow and low daytime temperatures as well as milder periods. For this reason, as part of the exploratory analysis of data during the original project (AW1402a), weekly variation in data were investigated in each group and no differences were found with respect to command use, dog behavior or training outcomes, suggesting weekly changes in environmental had minimal effects, and that trainers maintained consistent approaches to training over the weeks of data collection, despite the challenges of field conditions.

The second relates to differences in the general approaches to training between the three groups and in particular between Control Group 2 trainers and those in E-Collar and Control Group 1. Firstly, Control Group 2 appeared to primarily target recall training, with less time spent on other commands including sit, whereas the E-collar Group and Control Group 1 chose to work on both recall and general obedience including sitting (Table 3), perhaps indicating a greater focus on controlling the dog as well as achieving the target goal behavior. Furthermore, whilst the use of verbal signals was similar between the three groups, hand and in particular lead signals were less frequently used by Control Group 2 than either Control Group 1 or the E-collar Group; with Control Group 1 making more use of hand and lead signals during training than the E-collar Group. The use of multiple signals in training can have variable effects, with, for example, the use of additional contingencies such as lead pressure during a recall command, potentially affecting the rate of learning of the desired response. Improvement in learning would depend to some extent on the multiple signals being delivered consistently, and even then, dogs may form more reliable associations with some stimuli than others due to learning and perceptual biases or the nature of delivery. For example, it has been reported that visual signals during dog training may overshadow verbal ones when used at the same time (36). The explanation for differences in learning outcome may therefore lie in the degree to which dogs were exposed to rewarding and potentially aversive stimuli in the three groups and the range of signals used to guide the dogs' behavior.

Broadly speaking, dogs in Control Group 2 were asked to complete a recall task in response to verbal signals and normally received food reward(s) on return to trainer. Hand signals were rarely used and even though dogs were often on lead, lead pressure was very rarely recorded. As a consequence a single signal was used to cue the desired behavior and a single contingency (food) associated with successful completion of response. Similarly, training of a sit used a verbal “sit” command, with dogs receiving food reward once response had been completed. In summary, this group appeared to use the simplest and clearest contingencies for associative learning.

Dogs in the E-collar group were trained in accordance with industry best practice, with dogs' sensitivity to E-collar settings assessed early in training, and training focussed on associating the pre-warning cue, a collar born vibration, with exposure to the electric stimuli. In this way, the intensity of the electric stimulus could have been moderated to match the dog's tolerance and dogs could learn to modify their behavior to avoid exposure to the electric stimulus; a form of negative reinforcement. This sophisticated use of e-collars contrasts with that of some trainers reported in Cooper et al. (31), who used e-collars at their maximum settings and applied the electric stimulus after the dogs engaged in undesirable behavior, such as sheep chasing, without the use of the pre-warning cues. As buttons to deliver pre-warning cues were on same handset as the button for electric stimulus, it was not possible to reliably determine when electric stimuli were applied, so we should be cautious about inferring when stimuli were used during training schedules. For example, although one might predict that there would be more use of electric stimuli during early training as sensitivity is determined and an association formed between stimulus and desired response, or that electric stimuli would be more likely to be applied if the dog did not respond to initial command this cannot be verified from our data. For example, in previous published work (31), where vocalizations and abrupt changes in posture were recorded when dogs were remote from trainers, there was no evidence of change in frequency over 5 days of training. This freedom to adjust application of stimuli as part of the training program, as well as inclusion of other approaches to training the target or other behaviors, was consistent with the ethical approval of our project as well as our aim to assess best practice as advocated by the industry. Therefore, so long as dogs were not exposed to inescapable punishment, and trainers followed industry standards, we could not artificially impose standardized training programs, nor could we preclude trainers from using other signals and/or contingencies during training such as hand and lead signals. As a consequence, although we did not have the control over variables of experimental investigations of e-collar training [e.g., (37–39)], we did meet our aim of evaluating professional training of companion dogs with typically referred behaviors in the field.

Dogs in Control Group 1 were trained by the same trainers as the E-collar group and were expected to follow the same training approaches but without use of E-collar stimuli. Dogs in this group wore a de-activated dummy collar (as did dogs in Control Group 2) to control for the wearing of an unfamiliar device as well as part of the process of blinding observers to treatment in video analysis. As a consequence these dogs experienced collar fitting at start of each training session, but were not exposed to electric or vibration stimuli during training. These trainers therefore also used a mix of verbal, hand and lead signals, as the E-collar Group, but relatively few food rewards during training. It was also clear that the dogs received more lead and hand signals than the dogs in the E-collar group. Hand signals, involved not only hand gestures, but were also accompanied in some instances by physical contact with the dogs to gain their attention, stopping of ongoing behavior or pushing the dog into the desired position, whilst lead signals could be accompanied by what appeared to be sharp pulls on the lead. This more physical and potentially aversive use of contact or lead pressure was not observed in any of the videos relating to Control Group 2 but were clearly identified in both the E-collar Group and Control Group 1. These qualitative observations support the suggestion that the trainers involved in both the E-collar training and Control Group 1 were again more focussed on forcing compliance rather than shaping the desired response (40).

In summary, an important strategy within the reward-focused training of Control Group 2 was the positive reinforcement of successive approximations of the desired behavior, with mainly verbal signals, in order to build a strong contingency between command word and response (40). In contrast the E-collar group and Control Group 1 used a variety of signals and contingencies, including some potentially aversive handling and lead pressure during training. With good timing, these could result in negative reinforcement, although poor timing or imposition of the noxious stimuli in response to failure to perform the desired behavior would constitute a form of punishment. It has been frequently argued that the use of aversives in dog training results in poorer learning outcomes and poses greater welfare risks compared with largely reward based training (3–6). Our results demonstrate through direct evidence from real life situations, that the reward-focused training was, indeed, more efficient than methods which included potentially aversive stimuli such as electric stimuli or excessive lead pressure. Whilst our results may reflect general differences in training style of the trainer groups involved in the study rather than use of E-collar *per se*, we would argue that because the trainers who used E-collars were put forward by industry representatives as exemplars of best practice; their data (at least in relation to E-collar use) should be taken to represent a best case scenario for professional E-collar training. It is likely that less experienced trainers and owners would be less skilled and thus less effective in their use of the device [See (25, 35)].

Overall, the professional use of a reward-focused training regime, as demonstrated by Control Group 2, was superior to E-collar and Control Group 1 in every measure of efficacy where there was a significant difference. In addition, dogs in Control Group 1 showed no better learning outcomes than those in the E-collar group, indicating industry nominated trainers were as effective at modifying undesirable behavior, when they did not use e-collars as one of their training methods. Given the better target behavior response parameters associated with a reward-focused training programme, and the finding that the use of an E-collar did not create a greater deterrent for disobedience; we conclude that an E-collar is unnecessary for effective recall training. Given the additional potential risks to the animal's well-being associated with use of an E-collar (7, 25, 31, 38, 39), we conclude that dog training with these devices causes unnecessary suffering, due to the increased risk of a dog's well-being is compromised through their use, without good evidence of improved outcomes.

## Data Availability Statement

The raw data supporting the conclusions of this article will be made available by the authors, without undue reservation.

## Ethics Statement

The animal study was reviewed and approved by University of Lincoln Research Ethics Committee. Written informed consent was obtained from the owners for the participation of their animals in this study.

## Author Contributions

LC undertook video observation and behavioral coding of the training videos, as well as initial statistical analysis, and led the writing of the main article. JC and DM were LC's supervisors for her Master's thesis, providing support throughout. All authors made similar contributions to the final manuscript.

## Conflict of Interest

The authors declare that the research was conducted in the absence of any commercial or financial relationships that could be construed as a potential conflict of interest.
